# Clinical Manifestations and Modes of Death among Patients with Ebola Virus Disease, Monrovia, Liberia, 2014

**DOI:** 10.4269/ajtmh.17-0090

**Published:** 2018-02-05

**Authors:** Linda M. Mobula, Nathalie MacDermott, Clive Hoggart, Kent Brantly, William Plyler, Jerry Brown, Bev Kauffeldt, Deborah Eisenhut, Lisa A. Cooper, John Fankhauser

**Affiliations:** 1 Disaster Response Unit, Samaritan’s Purse, Boone, North Carolina;; 2 Division of General Internal Medicine, Department of Medicine, Johns Hopkins University, Baltimore, Maryland;; 3 Bloomberg School of Public Health, Johns Hopkins University, Baltimore, Maryland;; 4 Section of Paediatrics, Division of Infectious Diseases, Department of Medicine, Imperial College London, London, United Kingdom;; 5 Eternal Love Winning Africa (ELWA) Hospital, Monrovia, Liberia;; 6 SIM, Monrovia, Liberia

## Abstract

Although the high case fatality rate (CFR) associated with Ebola virus disease (EVD) is well documented, there are limited data on the actual modes of death. We conducted a retrospective, observational cohort study among patients with laboratory-confirmed EVD. The patients were all seen at the Eternal Love Winning Africa Ebola Treatment Unit in Monrovia, Liberia, from June to August 2014. Our primary objective was to describe the modes of death of our patients and to determine predictors of mortality. Data were available for 53 patients with laboratory-confirmed EVD, with a median age of 35 years. The most frequent presenting symptoms were weakness (91%), fever (81%), and diarrhea (78%). Visible hemorrhage was noted in 25% of the cases. The CFR was 79%. Odds of death were higher in patients with diarrhea (odds ratio = 26.1, *P* < 0.01). All patients with hemorrhagic signs died (*P* < 0.01). Among the 18 fatal cases for which clinical information was available, three distinct modes of death were observed: sudden death after a moderate disease process (44%), profuse hemorrhage (33%), and encephalopathy (22%). We found that these modes of death varied by age (*P* = 0.04), maximum temperature (*P* = 0.43), heart rate on admission (*P* = 0.04), time to death from symptom onset (*P* = 0.13), and duration of hospitalization (*P* = 0.04). Although further study is required, our findings provide a foundation for developing treatment strategies that factor in patients with specific disease phenotypes (which often require the use of aggressive hydration). These findings provide insights into underlying pathogenic mechanisms resulting in severe EVD and suggest direction for future research and development of effective treatment options.

## INTRODUCTION

The 2013–2016 West Africa Ebola virus disease (EVD) outbreak was unprecedented, given its duration, geographic scope, and high caseload of more than 28,600 reported cases and 11,300 deaths.^1^ In Monrovia, Liberia, faith-based organizations (Samaritan’s Purse and SIM) developed and operated the Eternal Love Winning Africa (ELWA-1 and 2) Ebola Treatment Units (ETUs) from June to August 2014, with the support of Medecins Sans Frontieres-Belgium (MSF-B).

During the period when the study was conducted, rapid spread of EVD occurred because of high population density in urban areas, unavailability of treatment units, and refusal of many ill people to seek care because of fear and superstition. In addition, fear prompted the surrounding community to arm themselves with machetes and protest in front of ELWA-2, demanding its closure in late July. This resulted in delays in developing the ELWA-3 ETU, which was intended to provide additional bed capacity. Limited knowledge of EVD led to challenges in recognizing the disease on the part of health-care workers, because of similarities in the presenting symptoms with endemic diseases such as malaria and typhoid fever. As a result, patients often presented in the late stages of EVD or died in the community without receiving appropriate treatment. Although caseloads continued to climb with limited bed capacity, the number of actors operating in Liberia remained extremely limited. ELWA-2 was the only functioning ETU in Monrovia at the end of July 2014.

To date, there has been no description in the literature of the predominant modes of death seen in the outbreak. Several studies have reported the clinical features of EVD and predictors of mortality. Studies from Guinea established an association between age, viremia, myalgia, hemorrhage, difficulty breathing, and mortality.^2–4^ A study from Sierra Leone emphasized that high viral load, severe hepatitis, and acute kidney injury are also predictors of mortality.^5^ Resource limitations, along with weak health systems in the affected countries, have to some degree prevented the determination of a comprehensive physiological mechanism of death. Instead, a descriptive approach to modes of death, defined as clinical characteristics observed in a patient leading to (and inclusive of) their terminal event, provides an opportunity to further understand the pathogenesis of the disease and guides areas for further research. In this article, we describe three distinct modes of death witnessed in a subset of our study population. A better understanding of predictors of death and the modes of death will assist with the development of improved clinical protocols and refine overall clinical care for patients with EVD. We conducted a retrospective, observational cohort study of clinical manifestation, modes of death, and predictors of mortality among confirmed cases of EVD admitted to ELWA-1 and ELWA-2.

## MATERIALS AND METHODS

The study protocol was approved by the Institutional Review Board at the University of Liberia and the Liberian Ministry of Health.

### Study site and patients.

Patients were seen at the ELWA-1 and ELWA-2 ETUs on the outskirts of Monrovia, Liberia, from June to August 2014. The ETU was run by faith-based organizations—Samaritan’s Purse and SIM—in partnership with the Liberia Ministry of Health and the nongovernmental organization MSF-B. The period covered in the study was a very difficult time in Monrovia, with patient numbers and needs far outnumbering medical staff, beds, and resources. To accommodate patients, the ELWA hospital chapel was transformed into a five-bed facility called the ELWA-1 ETU. ELWA-1 was soon replaced by ELWA-2, a 14-bed facility. The Liberian MOH had set up an ETU at John F Kennedy Hospital in Monrovia, but all the patients were transferred to ELWA-2 on its opening. ELWA-2 was the only functional ETU in Monrovia at that time. In the 24 hours after the opening of ELWA-2, the seven confirmed bed spaces were increased to 12, to accommodate the rapidly increasing number of patients. Within 5 days, 20 bed spaces had to be made, which necessitated significant reconfiguration of the ELWA hospital outpatient department, including the removal of entire walls. The influx of patients remained unabated, and soon, patients were required to lie on the floor to accommodate them. To make matters worse, because of a lack of burial teams, the ELWA-2 morgue was often overflowing.

A major limitation in providing care was the severe scarcity of trained staff, both national and international. A small group of international staff worked alongside Liberian staff, often working 12- to 14-hour shifts, 7 days in a row. Fear of contracting Ebola affected the recruitment of new staff, which was later exacerbated by infections among health-care workers at ELWA-2. Because of the condition of many patients at admission, large volumes of intravenous fluids were required. Because of the difficult circumstances mentioned earlier, it was not possible to provide the necessary volumes for all the patients, especially with the additional time constraints that resulted from the necessity of complex personal protective equipment.

All the patients included in the study were confirmed to have EVD by reverse transcription polymerase chain reaction (PCR) performed at the Liberia Institute of Biomedical Research laboratory outside of Monrovia.^6^ During this period, the laboratory did not provide cycle threshold values to the ETU, and this information is not traceable from the laboratory because of the difficulties in linking sample data to patients from this stage of the outbreak.

### Data collection.

Data were collected on exposure/occupational history, county of residence, vital signs, clinical signs and symptoms, PCR results, and outcomes for all patients admitted to the ETU (Tables 1 and 2). Patients were assessed at triage by a physician or physician’s assistant, then examined daily in the high-risk zone, where vital signs (temperature, heart rate, and respiratory rate) and physical examination findings were shared in real time with a scribe located in the low-risk zone. Although assessments were conducted for features of shock, such as capillary refill time and quality of pulse, this information was not consistently recorded and, therefore, cannot be interpreted from the data set. Available resources at that time did not allow for clinical laboratory testing, including monitoring of electrolytes.

**Table 1:** Baseline and clinical characteristics of patients with confirmed Ebola virus disease at ELWA-1 and ELWA-2

Characteristic	Result
Median age, years (IQR)	35 (28.5–45)
Male:female ratio	28:25 (1.1:1)
Case fatality rate	79.2%
Median duration of hospitalization, days (IQR)	5 (3–9)
Median days between symptom onset and admission (IQR)	5.5 (3–7)
Reported exposure	
Nosocomial	16 (28%)
Household	15 (26%)
Family member of health-care worker	5 (9%)
Funeral	4 (7%)
Church (hands-on praying of patients by pastor)	1 (2%)
Assisted with patient transport	1 (2%)
Market contact	2 (4%)
Not reported/unknown	13 (23%)
County of residence	
Montserrado	33 (62%)
Bomi	10 (19%)
Bong	7 (13%)
Margibi	1 (2%)
Grand Bassa	1 (2%)
Unknown	1 (2%)
Signs and symptoms	
Weakness	40/44 (91%)
Fever	39/48 (81%)
Diarrhea	35/45 (78%)
Nausea	32/45 (71%)
Vomiting	27/45 (60%)
Abdominal pain	22/41 (54%)
Headache	21/40 (53%)
Conjunctival infection/subconjunctival hemorrhage	13/44 (30%)
Visible hemorrhage	11/44 (25%)
Respiratory distress	9/44 (21%)
Hiccups	2/44 (5%)
Vital signs on admission (median/IQR)	
Temperature (°C)	37.2 (36.8–38.2)
Heart rate (beats per minute)	88 (80–102)
Respiratory rate (breaths per minute)	22 (20–28)
Maximum temperature during admission (°C)	38.4 (37.9–39.4)

ELWA = eternal love winning Africa; IQR = interquartile range. *N* = 53.

**Table 2:** Characteristics of survivors and nonsurvivors

Characteristic	Survivors *N* = 11	Nonsurvivors *N* = 42	Unadjusted OR (95% CI)	*P* value	Adjusted OR (95% CI)	*P* value
Median age (IQR)	40 (23–43)	35 (29.8–48.2)	–	0.63	–	–
Male:female ratio	1.8:1	1.9:1	1.1 (0.2–5.3)	1	1.0 (0.2–4.8)	1
Health-care worker	4/11 (36.4%)	14/41 (34.1%)	0.9 (0.2–5.0)	1	1.0 (0.2–6.2)	1
Median duration of hospitalization in days (IQR)	17 (13.5–20.5)	4 (2.3–6)	–	3 × 10^−6^	–	–
Duration of hospitalization > 10 days*	9/11 (81.8%)	1/42 (2.4%)	136 (12–7,650)	1 × 10^−7^	124 (10.6–7,380)	2 × 10^−7^
Median days (IQR) between symptom onset and admission	4.5 (2.3–6.8))	5 (3–7)	–	0.67	–	–
Signs and symptoms						
Weakness	8/9 (88.9%)	32/35 (91.4%)	1.3 (0.0–19.3)	1	1.0 (0.0–16)	1
Fever	8/11 (72.7%)	31/37 (83.7%)	1.9 (0.3–11.6)	0.41	1.8 (0.2–10.5)	0.67
Diarrhea	2/9 (22.2%)	33/36 (91.7%)	32.8 (4.1–464)	8 × 10^−5^	26.1 (3.5–332)	2 × 10^−4^
Nausea	6/9 (66.7%)	26/36 (72.2)	1.3 (0.2–7.6)	0.7	1.3 (0.2–7.8)	0.7
Vomiting	4/9 (44.4%)	23/36 (63.9%)	2.2 (0.4–13.1)	0.45	2.1 (0.4–12.7)	0.45
Abdominal pain	4/9 (44.4%)	18/32 (56.2%)	1.6 (0.3–9.6)	0.71	1.42 (0.2–8.8)	0.72
Headache	3/8 (37.5%)	18/32 (56.2%)	2.1 (0.3–15.9)	0.44	2.4 (0.4–17.7)	0.43
Hemorrhagic symptoms:						
Visible†	0/10 (0%)	11/34 (32.4%)	‡	0.046	‡	0.041
Subconjunctival†	0/10 (0%)	13/34 (38.2%)	‡	0.021	‡	0.019
Combined	0/10 (0%)	17/34 (50.0%)	‡	3.7 × 10^−3^	‡	2.9 × 10^−3^
Respiratory distress	2/10 (20%)	7/34 (20.6%)	1.0 (0.2–12.2)	1	1.1 (0.2–12.8)	1
Hiccups	0/10 (0%)	2/34 (5.9%)	‡	1	‡	1
Vital signs						
Median temperature (°C) on admission (IQR)	38 (37–38.6)	37.2 (36.8–37.9)	–	0.26	–	–
Median maximum temperature (°C) during admission (IQR)	38.4 (38–39.2)	38.6 (38–39.4)	–	0.81	–	–
Median admission heart rate (IQR) (beats per minute)	77 (70–84)	88 (82–102)	–	0.1	–	–
Median admission respiratory rate (IQR) (breaths per minute)	27 (25–30)	22 (20–27)	–	0.23	–	–

CI = confidence interval; IQR = interquartile range; OR = odds ratio.

* 10 days is the midpoint in the median duration of hospitalization between those who died and those who survived.

† Visible hemorrhagic symptoms refer to active bleeding from one or more sites; subconjunctival refers to the severe conjunctivitis several patients presented with, some of whom progressed to frank subconjunctival hemorrhage.

‡ All patients with these symptoms died, resulting in an infinite OR.

### Evaluation of mode of death.

Reporting the mode of death was done in situations where the patient’s clinical condition had been consistently witnessed by the clinical team preceding death. In addition, it was included in the case of sudden death when the patient had been witnessed to be stable, alert, and mobile shortly before death, without signs of profuse hemorrhage. As such limited records are available (*N* = 18), the mean characteristics of which are presented in Table 3. The modes of death described were not determined in advance. We describe three distinct modes of death, as directly witnessed by the ELWA-2 clinical team leader. As staffing levels at the ETU were inadequate, all modes of death evaluations were made by a single observer. Postmortem examination was not possible. The following modes of death were observed:

1. Profuse hemorrhage
  Occurring 5–7 days from symptom onset, death ensued from a terminal hemorrhagic event within 24 to 48 hours of onset of frank hemorrhage.
2. Encephalopathy
  Commencing 7–8 days after symptom onset, a reduced conscious level (responsive only to pain) developed, with a marked pyrexia (> 39.4°C) and tachypnea that progressed to Kussmaul breathing, with rapid deterioration and death within approximately 48 hours of reduced conscious level.
3. Sudden death
  Sudden death is defined as a sudden, unexpected, and immediate terminal event, which was not overtly hemorrhagic in nature, following a moderate illness of approximately 10–12 days. In most cases, this terminal event presented as a seizure-like event or sudden collapse in an ambulating patient.

**Table 3:** Characteristics of modes of death

Characteristic	Mode of death		
	Hemorrhagic	Encephalopathy	Sudden death
Time to death from symptom onset (days): median IQR)	8 (7.25–9.25)	10 (9.5–11)	12 (9.5–13.5)
*P* value*	0.13	1	0.17
Age (years): median (IQR)	27 (26–30)	47 (37.3–57)	33 (31.5–45)
*P* value*	0.045	0.041	0.92
Maximum temperature (°C): median (IQR)	38.6 (37.8–38.8)	39.1 (38.1–40.1)	38.6 (38.1–39.4)
*P* value*	0.67	0.43	0.77
Admission heart rate (beats per minute): median (IQR)	84 (80–104)	100 (94–108)	85 (83–88)
*P* value*	0.65	0.067†	0.24
Onset of symptoms to hospitalization (days): median (IQR)	4 (3.75–4.75)	6 (5–6.5)	5 (4–6.5)
*P* value*	0.42	0.47	0.90
Duration of hospitalization (days): median (IQR)	4 (4–5)	4.5 (3.75–5)	8.5 (5.5–9.25)
*P* value*	0.22	0.27	0.041

IQR = interquartile range.

* *P* value for the comparison of the individual mode of death vs. the other two modes of death combined.

† When an individual comparison is made between the encephalopathy mode of death and sudden death, the *P* value for admission heart rate falls to 0.045.

### Treatment.

All patients received treatment according to standard MSF protocols, including oral rehydration solution, intravenous antibiotics, antimalarial therapy, vitamins A and C, and analgesia and sedation when required. All patients with poor oral intake or heavy fluid losses received intravenous fluids. However, because of limitations in accessing patient charts, the volumes administered cannot be reliably reported.

### Variables.

We evaluated mortality among patients with EVD as a binary variable—death in the ETU or survival. Independent variables evaluated included the following binary variables: gender and presence or absence of symptoms (on admission and daily). The following continuous variables were evaluated: time at onset of symptoms to admission, duration of hospitalization, and vital signs on admission.

### Statistical analysis.

Mode of death and patient characteristics were summarized using descriptive statistics. *P* values for differences of binary characteristics between survivors and nonsurvivors were calculated using Fisher’s exact test, and unadjusted odds ratios (ORs) for the effect of the characteristic on mortality were calculated directly from 2 × 2 contingency tables. ORs and *P* values adjusted for age older than or younger than 40 years were calculated using the Cochran–Mantel–Haenszel exact test. *P* values for differences in continuous characteristics between modes of death and survivors and nonsurvivors were calculated using the nonparametric Kruskal–Wallis test. For continuous covariates, ORs were only reported for those that were statistically significant; in our analyses, this was found to be duration of hospitalization. The OR was calculated for binary characteristics by defining a binary covariate, setting the duration of hospitalization at a threshold, calculated as the midpoint between the medians in survivors and nonsurvivors. The Breslow–Day test was used to identify differential effects of age older than or younger than 40 years. Hypothesis tests were two tailed, with a *P* value of less than 0.05 indicating statistical significance. All statistical analyses were performed with *‘R’ Language and Environment for Statistical Computing (R) 3.2.2*.

## RESULTS

### Baseline characteristics.

Of the 69 patients admitted to the ELWA-1 and ELWA-2 ETUs from June to August 2014, 53 (77%) were confirmed EVD cases. Of the remainder, five had no determinable result, three were not tested, and eight tested negative for EVD. The three who were not tested were either deceased on arrival or died within a few hours of admission.

Table 1 describes the basic patient characteristics and symptom incidence. A comparison of characteristics of patients who survived versus those who died is shown in Table 2. Denominator figures may vary because of missing data.

**Figure 1.**
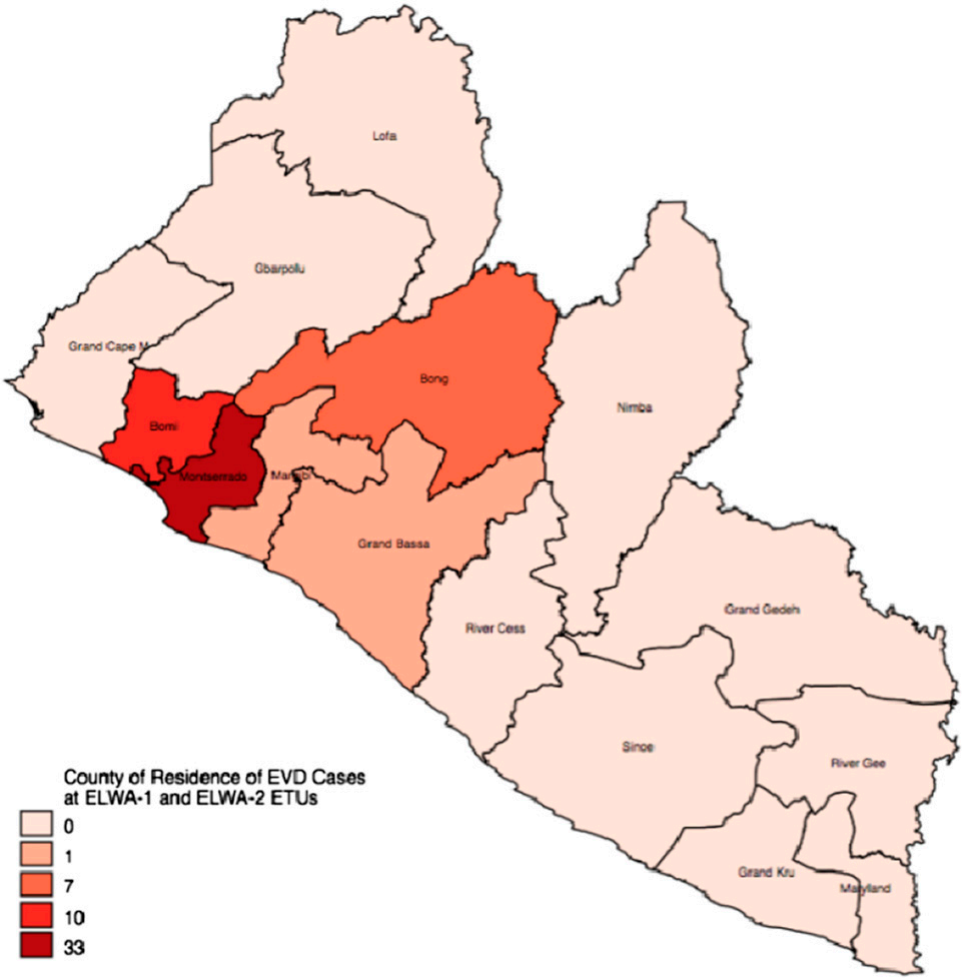
Map of county of residence of confirmed Ebola virus disease (EVD) cases. This figure appears in color at www.ajtmh.org.

The majority of EVD-positive patients admitted to ELWA from June to August 2014 resided in Montserrado County, although patients were also admitted from Bomi, Bong, Margibi, and Grand Bassa counties (Figure 1). The primary mode of transmission was nosocomial, with 28% of patients reporting nosocomial transmission, followed by direct contact with a household member (26%).

The median age of patients was found to be 35 years (Interquartile range [IQR] = 28.5–45); the median age among nonsurvivors was lower (35 years, IQR = 29.8–48.2) than that among survivors (40 years, IQR = 23–43) but was not statistically significant (*P* = 0.63). The case fatality rate (CFR) was 79% among all patients (Table 1).

The most common symptom was weakness (91%), followed by fever (81%) and diarrhea (78%). In comparison with other data sets, our patients had a relatively high incidence of hemorrhagic signs at 38.6%. An absence of fever was noted among 18.8% of all laboratory-confirmed Ebola patients on admission (Table 1). Although the temperature range on admission was broad (35.6– 39.2°C), only one patient presented with clinical hypothermia; this patient died within a few hours of admission.

### Modes of death.

**Figure 2.**
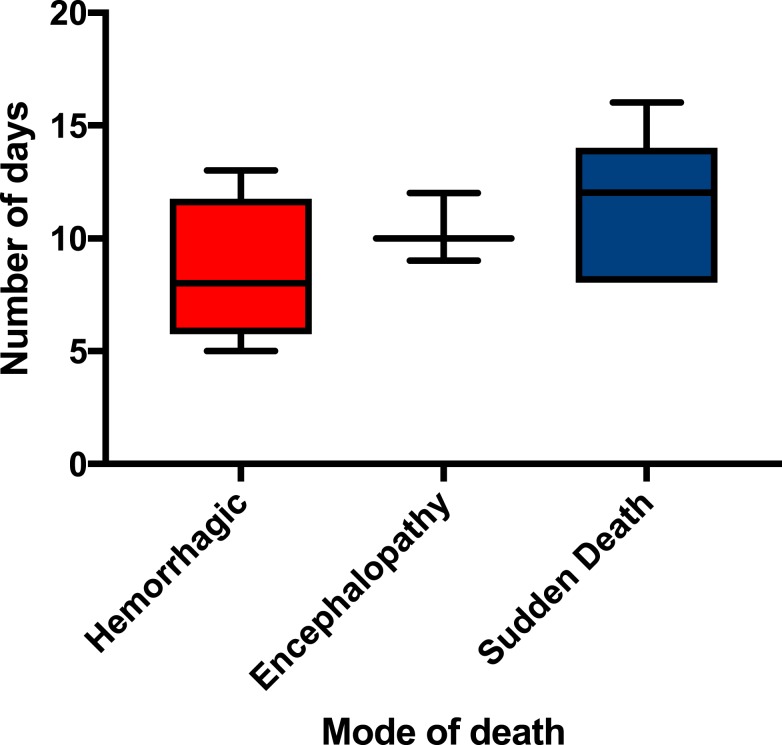
Time to death from symptom onset compared with mode of death in Ebola virus disease. This figure appears in color at www.ajtmh.org.

**Figure 3.**
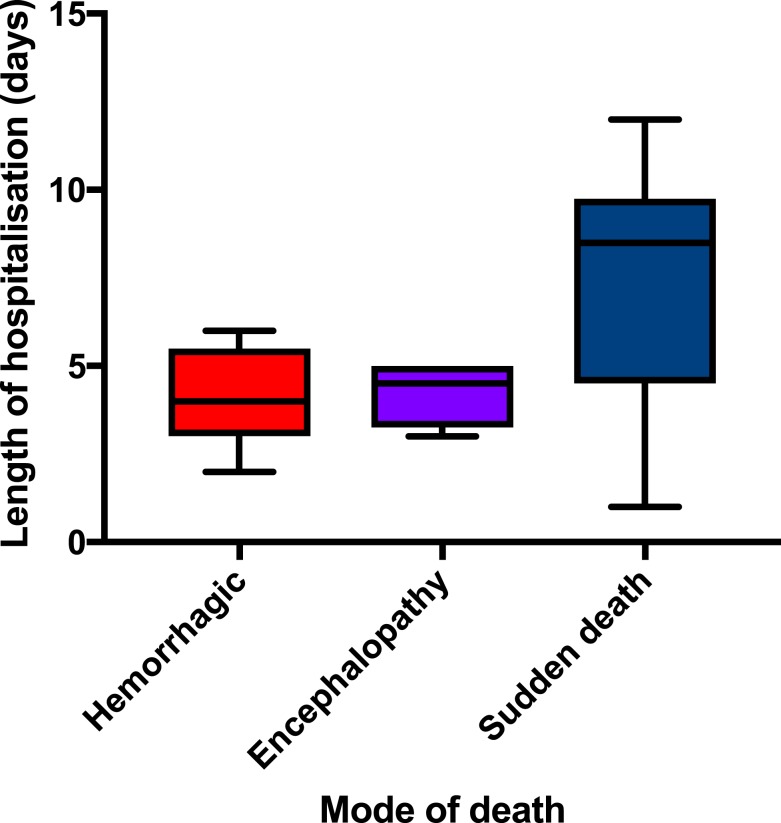
Duration of hospitalization by mode of death. This figure appears in color at www.ajtmh.org.

When analyzed, the modes of death described previously revealed differences in the age of the patients, the maximum temperature, the time to death from symptom onset, and the heart rate on admission (Table 3). The median time to death values were not statistically significant, but orientation of values is demonstrated in Figure 2. For profuse hemorrhage, the median time to death was 8 days; for encephalopathy, it was 10 days; and for sudden death, it was 12 days. Figure 3 demonstrates that the variation in time to death is predominantly due to the patient’s duration of hospitalization as opposed to the length of time between onset of symptoms and admission to the ETU.

**Figure 4.**
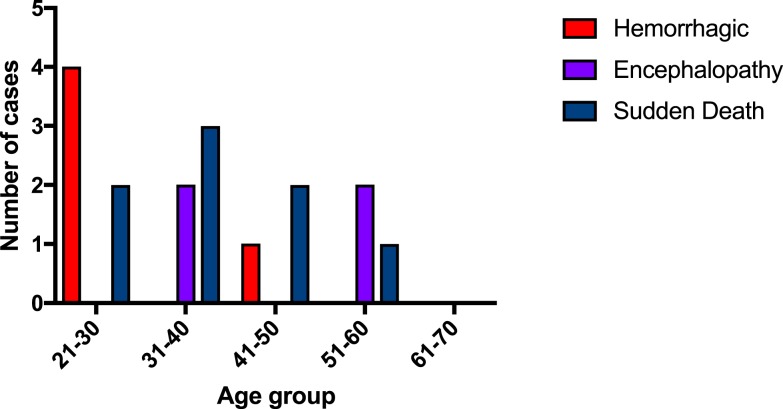
Relationship of age to mode of death in Ebola virus disease. This figure appears in color at www.ajtmh.org.

Patients who died of profuse hemorrhage were generally younger (Figure 4), with a median age of 27 years (IQR = 26–30), which was significantly different (*P* = 0.045) from the other two modes of death combined: encephalopathy median age of 47 years (IQR = 37.3–57) and sudden death median age of 33 years (IQR = 31.5–45). The median age of 47 years for the encephalopathy mode of death was also significantly higher than that of the other two modes of death combined (*P* = 0.041). The distribution of age by mode of death is shown in Figure 4.

**Figure 5.**
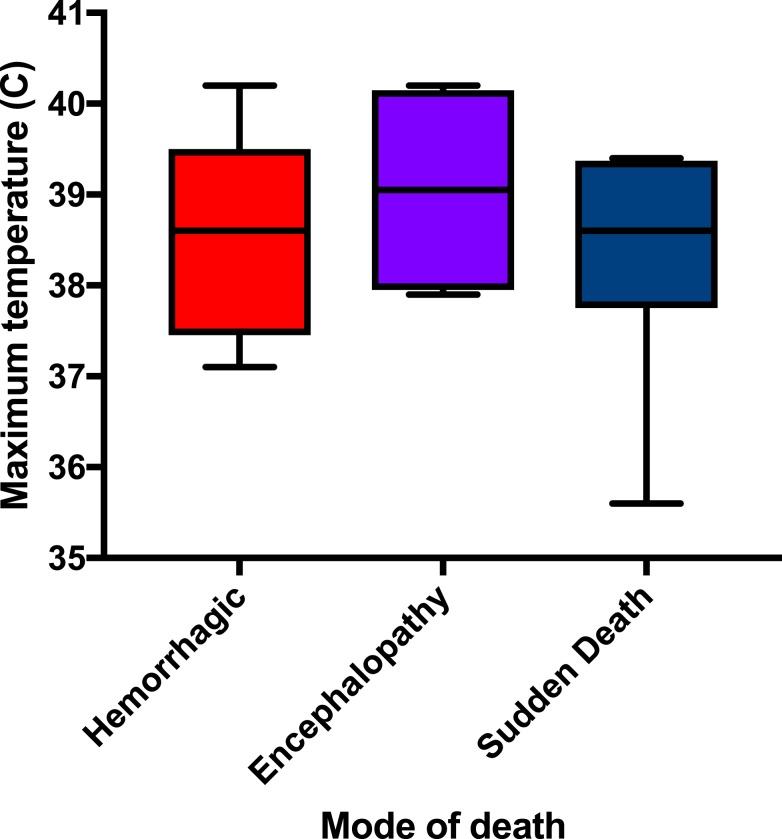
Maximum temperature distribution by mode of death in Ebola virus disease. This figure appears in color at www.ajtmh.org.

**Figure 6.**
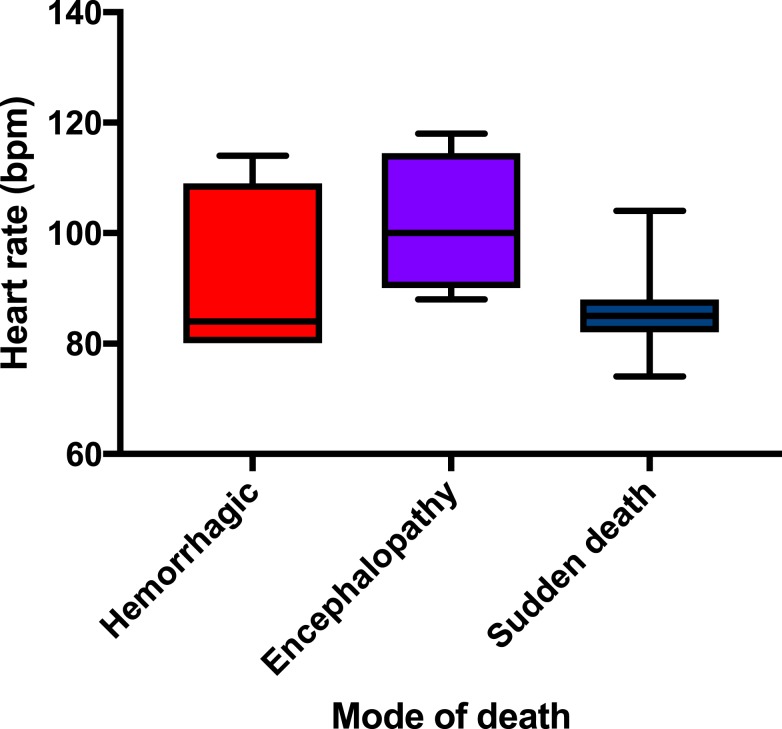
Admission heart rate distribution by mode of death in Ebola virus disease. This figure appears in color at www.ajtmh.org.

Those in the hemorrhagic category had a tendency to have a lower Tmax (median 38.6°C, IQR = 37.8–38.8) than those in the other categories but demonstrated the same median temperature as those in the sudden death category (38.6°C, IQR = 38.1–39.4). Those with encephalopathy demonstrated a higher Tmax (median 39.1°C, IQR = 38.1–40.1); this mode of death had clinical features that could be consistent with a cytokine storm. These differences were not statistically significant, but the ranges are demonstrated in Figure 5. Of note, the median heart rate on admission was significantly lower (*P* = 0.045) for the sudden death category (85 beats per minute, IQR = 83–88) when compared with that in the encephalopathy mode of death (100 beats per minute, IQR = 94–108) as demonstrated in Figure 6.

When compared with survivors, there was unsurprisingly a significant difference between modes of death and duration of hospitalization (*P* < 0.01 for all modes of death), as demonstrated in the comparison of survivors and deceased patients shown in Table 2. The only other variable that significantly differed from survivors (*P* = 0.043) was between the median admission heart rate in the encephalopathy mode of death (100 beats per minute, IQR = 94–108) and the median admission heart rate of survivors (77 beats per minute, IQR = 70–84).

### Association between diarrhea, hemorrhage, and mortality.

Patients with diarrhea had higher odds of death (OR = 26.1, 95% confidence interval = 3.5–332, *P* < 0.01) compared with those without diarrhea. All patients who exhibited any type of hemorrhagic symptom died, although the terminal event for a proportion of these patients was not profuse hemorrhage; some had a more moderate disease process terminating in a sudden death event that was not overtly hemorrhagic. Other patients demonstrated overt hemorrhagic symptoms that progressed to a profuse and ultimately terminal hemorrhagic event (profuse hemorrhage mode of death). Where records are available, those patients with mild hemorrhagic symptoms demonstrated a moderate disease process which terminated with a sudden death event (sudden death mode of death). Patients in the encephalopathy mode of death demonstrated hemorrhagic conjunctivitis but did not demonstrate signs of overt bleeding.

### Treatment.

Comprehensive data on the volume of intravenous fluids that patients received are not available. Documentation of whether fluid administration was given is available for 33 patients. Of these 33 patients, 30 (91%) received intravenous fluids. Of the three who did not, one died at triage before obtaining intravenous access, one declined intravenous fluids (this patient went on to survive), and the third patient demonstrated minimal symptoms for only 24 hours, despite a contact history and positive EVD PCR; this patient also survived.

## DISCUSSION

A stark finding from this small data set is the well-defined modes of death and clinical syndrome patients demonstrated in the 24 to 48 hours before death and the suggestion that these differed with age and duration of hospitalization, but not time from symptom onset to presentation at a treatment facility. There could be several reasons for this including a slightly different underlying pathogenesis or interaction between the immune system and virus at different ages. Genetic predisposition dictating immune response may be another possible explanation for the varied modes of death observed and is currently under investigation. Given the evidence for minimal viral sequence variation during the epidemic, varied pathogenicity of the infecting virus is unlikely.^7,8^

### Modes of death.

The modes of death described suggest potential mechanisms of pathogenesis leading to death. This may indicate that earlier clinical interventions might be appropriate in the management of cases, such as early identification and aggressive management of coagulopathy. Although these categories are not documented elsewhere in the literature, written correspondence with colleagues present during the early stages of the epidemic in all three heavily affected nations highlighted similar categories. This should be studied in more detail as understanding these mechanisms may help to further prioritize treatment of those likely to deteriorate more rapidly.

The sudden death category is of particular interest as these patients suffered a longer and more moderate illness when compared with the other two categories, with a significantly longer hospital admission (*P* = 0.041), but no difference in the time to admission from symptom onset (*P* = 0.90). These patients often appeared as if they may survive yet experienced a sudden event that resulted in death. Although some suffered a terminal seizure event, the cause of this remains unclear. This may have been from direct viral invasion of neural tissue or the consequence of intracranial hemorrhage, vaso-occlusive events, electrolyte disturbances, or cardiac arrhythmias. Those patients who did not suffer a witnessed seizure event were simply found collapsed, usually while ambulating. It is unclear if a seizure had occurred. This group of patients did not display clear symptoms of encephalitis, but some demonstrated a mild hemorrhagic tendency such as bleeding around IV line sites. Interestingly, many of those who died of a sudden death event were found to be in full rigor mortis when death was being certified, which was often within an hour of death, if not less than 30 minutes of death.

All patients who died of profuse hemorrhage had frank hemorrhagic signs in the 24 to 48 hours before death, often from multiple locations, which gradually became more pronounced in the lead up to a final profuse hemorrhagic event. Many patients who went on to have a terminal hemorrhagic event were noted to become afebrile approximately 24 to 48 hours before the onset of frank hemorrhagic signs. This may reflect defervescence before a profuse hemorrhagic event. A similar period of defervescence has been noted in dengue shock syndrome shortly before the onset of shock^9–11^ and may reflect decompensated shock in patients with EVD also.

Some patients had mild hemorrhagic signs, such as bleeding around intravenous line sites, melena, or metrorrhagia. However, their death did not result from an obvious profuse, terminal hemorrhagic event. The majority of these patients presented a picture consistent with sudden death, as their disease process was moderate and more prolonged, and their terminal event was a collapse or a seizure-like event. It cannot be determined whether they suffered a sudden intracranial hemorrhagic event, but this must be given due consideration. The patients in the encephalopathy category were not noted to have any overt hemorrhagic symptoms other than conjunctivitis.

The patients in the encephalopathy mode of death presented with clinical features similar to a cytokine storm with hyperpyrexia, encephalopathy, and features of acidosis such as tachypnea with Kussmaul breathing. It is interesting that these patients seemed to be in an older age category (Figure 4) and were more likely to be tachycardic at admission (Figure 6) when compared with other modes of death and with survivors. It may be that they had a more profound cytokine production in response to infection than other modes of death, which may explain a more rapid deterioration to shock and then death. However, diffuse proinflammatory cytokine production may also be related to the frank hemorrhage^12^ noted in the hemorrhagic mode of death.

### Predictors of mortality.

In the analysis of clinical symptoms and outcomes, diarrhea was a significant predictor of death (OR = 26.1, *P* < 0.01). These findings are similar to those of other studies that have suggested that intravascular volume depletion likely leads to worse outcomes among patients with EVD.^3–5,13^ For this reason, interventions such as aggressive oral and intravenous rehydration are recommended in the early stages of disease for patients with EVD.^3–5,13^ Although approximately 90% of our patients received intravenous fluid, it was not possible to reliably assess the impact of this from the current data set. Although some patients may have received adequate fluid resuscitation, some patients with EVD require in excess of 10 L of intravenous fluids per day to meet their ongoing losses.^13,14^ It was not possible to administer this level of fluid resuscitation because of the extreme staffing constraints, number of patients, and lack of available beds.

We reported hemorrhagic signs in 38.6% of our patients, which is similar to a study from Guinea but higher than what has been reported elsewhere.^4,5,15^ Ascertaining whether patients had melena can be difficult, which may account for the lower rates of hemorrhagic disease reported in other study cohorts. It may also be that milder cases of EVD did not present to the ETU for treatment, given the circumstances at the time, resulting in the population of this study being more representative of those with severe disease.

Triage processes that distinguish severity of illness should take into account the presence of either diarrhea or hemorrhage on admission, given the higher likelihood of death and greater needs with regard to clinical and hygiene management. Similar to a study in Sierra Leone, we found that a moderate proportion of patients with confirmed EVD presented without a fever at the time of admission.^16^ This finding has repercussions regarding screening and triage protocols, which may need to be reevaluated.

In April 2015, the World Health Organization reported CFRs in Liberia to be 42%, a significant decline from CFRs at ELWA in June to August estimated at 79% (Table 1). CFRs at the onset of the outbreak in Liberia in June to August were higher than those in neighboring Guinea (60%) but consistent with CFRs previously reported for EBOV outbreaks.^17–19^ A recent study in *Cell* found that an Ebola glycoprotein mutant (GP-A82V) arising early during the West African epidemic increased infectivity of human cells and may have contributed to increased mortality in Liberia and Sierra Leone. The authors of this study found that both viral load and GP-A82V were significantly associated with increased mortality.^20^ This mutation may have led to increased viremia among cases in Monrovia, which has been shown to account for increased CFRs.^2^

In our study cohort, the fatal cases had a median number of hospitalization days of 4, and 52% died within 4 days of being admitted. Although there was no significant association between the interval of symptom onset and ETU admission and mortality, this is contrary to a study in Sierra Leone where such an association was found.^16^ Our study was likely underpowered to find such an association. The decline in the CFR as the epidemic evolved was likely due to multiple factors including a decrease in the time between symptom onset and hospitalization. This may have resulted in lower viremia at the time of admission. Previous studies suggest that lower viremias at the time of admission are associated with more favorable outcomes.^2,5^ Improved training of health-care workers and better staff-to-patient ratios in treatment facilities likely also contributed.

### Limitations.

Our study had several limitations. The small sample size will have affected the power to determine significant differences and associations. Because of limitations in laboratory diagnostics and imaging, it was not possible to determine the exact mechanism of death, only to conduct careful observations of the mode. As the mode of death was noted by a single observer, this introduces an observer bias but does avoid interobserver variability. In the future, access to basic biochemistry and acid–base balance data on patients would help to determine, or at least exclude, certain mechanisms of death. More detailed laboratory diagnostics would have also provided further insights into the association between factors such as anemia, thrombocytopenia, electrolyte deficiencies, renal failure, transaminitis, acidosis, coagulopathy, and outcomes.

In conclusion, we describe three distinct modes of death that patients with EVD succumbed to. This highlights the need for better understanding of the mechanisms of death in EVD to provide further insights into the pathogenesis and complications of the disease. We also demonstrate a strong association between diarrhea, hemorrhage, and mortality, suggesting that more aggressive hydration and a higher level of care for patients presenting with diarrhea or hemorrhage may improve outcomes.
